# Virtual Reality Therapy for the Management of Chronic Spinal Pain: Systematic Review and Meta-Analysis

**DOI:** 10.2196/50089

**Published:** 2024-02-12

**Authors:** Tongtong Zhang, Xin Li, Xuan Zhou, Lixia Zhan, Fan Wu, Zefan Huang, Yuxun Sun, Yufei Feng, Qing Du

**Affiliations:** 1 Department of Rehabilitation Xinhua Hospital Affiliated to Shanghai Jiao Tong University School of Medicine Shanghai China; 2 School of Exercise and Health Shanghai University of Sport Shanghai China; 3 The Second People’s Hospital of Beihai Beihai China; 4 College of Rehabilitation Sciences Shanghai University of Medicine & Health Sciences Shanghai China; 5 Chongming Hospital Shanghai University of Medicine & Health Sciences Shanghai China

**Keywords:** virtual reality, chronic spinal pain, inflammation-related pain, systematic review, meta-analysis

## Abstract

**Background:**

The effectiveness of virtual reality (VR) therapy in adults with chronic spinal pain (CSP) is unclear.

**Objective:**

This study was conducted to compare the effectiveness of VR therapy and other therapies in adults with CSP, especially patients with inflammation-related pain.

**Methods:**

PubMed, Web of Science, Cochrane Library, Embase, and CINAHL databases were searched up to November 11, 2023. Randomized controlled trials (RCTs) comparing adults with CSP receiving VR therapy with those receiving other therapies were included. The trial registration platform as well as the reference lists of included studies and previous systematic reviews and meta-analyses were manually searched. Two independent reviewers performed study selection, data extraction, risk-of-bias assessment, and evaluation of the quality of the evidence. The weighted mean difference (WMD) was used as the effect size used to synthesize the outcome measure.

**Results:**

In total, 16 RCTs involving 800 participants were included in this meta-analysis. The pooled data from 15 (94%) RCTs including 776 (97%) participants showed that VR therapy was superior in improving pain intensity (WMD=–1.63, 95% CI –2.11 to –1.16, *P*<.001, *I*^2^=90%) and reducing inflammatory markers, including C-reactive protein (WMD=–0.89, 95% CI –1.07 to –0.70, *P*<.001, *I*^2^=0%), tumor necrosis factor-alpha (WMD=–6.60, 95% CI –8.56 to –4.64, *P*<.001, *I*^2^=98%), and interleukin-6 (WMD=–2.76, 95% CI –2.98 to –2.53, *P*<.001, *I*^2^=0%). However, no significant differences were found in terms of the spinal range of motion (ROM), disability level, or fear of movement. In addition, 10 (63%) of the included RCTs had a high risk of bias.

**Conclusions:**

VR therapy may be an effective and safe intervention for reducing symptoms in patients with CSP, as it is shown to exert significant analgesic effects and beneficial improvements in inflammatory factor levels. However, this approach may not have significant effects on the spinal ROM, disability level, or fear of movement. Notably, the quality of the evidence from the RCTs included in this study ranged from moderate to low. Therefore, we recommend that readers interpret the results of this study with caution.

**Trial Registration:**

PROSPERO CRD42022382331; https://www.crd.york.ac.uk/prospero/display_record.php?RecordID=382331

## Introduction

Chronic spinal pain (CSP), which most commonly includes chronic low back pain (CLBP) and chronic neck pain (CNP), is the leading cause of years with disability worldwide [1,2] and constitutes the most frequent reason for patients to seek medical care in any given year. The lifetime prevalence of low back pain (LBP) is 84%; more specifically, the lifetime prevalence of CLBP is 23%, and LBP accounts for approximately 11%-12% of cases of disability [3]. CSP is recognized as a biopsychosocial syndrome [4]. Prolonged pain can lead to anxiety, depression, and other negative emotions and is particularly significant in patients with CSP, as it is associated with decreased quality of sleep and reduced physical activity, thus placing tremendous strain on health care systems and world economies [5].

Previous studies have reported that an intervertebral disc undergoes aging or pathological changes in the adjacent region in patients with CSP, exposing cells within the nucleus pulposus to macrophages, resulting in an inflammatory response that might trigger pain [6,7]. The guidelines recommend that nonsteroidal anti-inflammatory drugs (NSAIDs) be the primary choice for patients with chronic pain [8]. However, compared with a placebo, NSAIDs can reduce CSP by controlling the level of inflammation but do not achieve clinically important efficacy [9]. Additionally, long-term use may be associated with adverse effects (eg, gastrointestinal reactions, hepatic and renal damage, and cardiovascular risk) [10]. Several studies have shown that conventional nonpharmacological therapies, such as spinal manipulation, acupuncture, exercise therapy, yoga, and cognitive-behavioral therapy, are beneficial for reducing CSP and improving psychological symptoms but have limited effects (small to moderate) [11-14]. Effective cognitive-behavioral therapies are not widely accessible due to the reliance on therapist experience, and the long-term effectiveness of these therapies remains unclear [15]. Notably, the majority of patients with CSP have goals of pain management (using ongoing care) rather than “curing” (care with a specific end) for their therapeutic care because of the complexity of the causes of chronic pain [16]. Thus, pain management is as important as the control of inflammation levels for patients with CSP. There is an urgent need for an alternative analgesic nonpharmacological and anti-inflammatory strategy for patients with CSP.

Virtual reality (VR) is typically characterized by low cost, easy availability, reusability, and personalized customization; VR therapy has been used as an alternative approach for pain management in various populations, such as individuals with spinal cord injuries, burns, and phantom limb pain [17-19]. VR can be categorized into 2 types: nonimmersive virtual reality (NIVR) and immersive virtual reality (IVR). NIVR is managed using a computer or console gaming system and a 2D interface device (mouse, keyboard, or gamepad, joystick), and patients do not need to be fully immersed in a virtual environment for experience [20].With the use of professional equipment, hardware, and configuration of the corresponding software, IVR can mimic reality by enabling the user to interact with the virtual environment [21]. A recent study demonstrated that regular exercise with the use of VR might be related to a decrease in inflammation in participants undergoing chronic hemodialysis [22], and inflammatory arthritis–targeting innovative teaching approaches based on VR technology are considered feasible [23]. There is limited evidence regarding the beneficial effects of VR therapy on pain in patients with CNP [24] and CLBP [25,26]; furthermore, there is insufficient focus on inflammatory factors. Therefore, this study aimed to investigate the potential efficacy of VR in reducing pain intensity and the levels of inflammatory factors in patients with CSP, thereby providing an updated summary of the existing evidence.

## Methods

### Study Protocol and Registration

This systematic review and meta-analysis was conducted in accordance with the Preferred Reporting Items for Systematic Reviews and Meta-Analysis (PRISMA) guidelines. The PRISMA checklist is given in Multimedia Appendix 1. The study protocol was registered in the PROSPERO database (CRD42022382331). The *Cochrane Handbook for Systematic Reviews of Interventions* (version 5.1.0) was followed [27].

### Search Strategy

#### Search Sources

PubMed, Web of Science, Cochrane Library, Embase, and CINAHL electronic databases were searched from inception to November 11, 2023, to identify relevant studies. The reference lists of the included studies, as well as systematic reviews and meta-analyses that examined the efficacy of VR in patients with CSP, were manually searched for additional eligible studies. The trial registration platform ClinicalTrials was also searched for ongoing studies that reported sufficient data on the efficacy of VR for CSP.

#### Search Terms

The studies on VR for CSP were identified by formulating appropriate search terms. These terms were selected based on the target population (spinal pain, neck pain, thoracic pain, back pain, LBP, sacral pain, and intervertebral disc pain), target intervention (eg, VR), and target study design (eg, randomized controlled trial [RCT]). The detailed search strategy is shown in Multimedia Appendix 2.

### Study Eligibility Criteria

The inclusion criteria were as follows:

- Participants: adults older than 18 years with chronic pain (more than 12 weeks) in the spinal region were included, except those who were receiving analgesic medication and who had cancer-related pain or neuropathic pain (eg, neuropathic pain after spinal cord injury, herniated disc with compression, sciatica, or lumbosacral radiculitis).
- Intervention: VR therapy.
- Comparisons: sham stimulation, usual care, and conventional treatment.
- Outcomes: pain intensity, inflammatory markers (eg, C-reactive protein [CRP], tumor necrosis factor-alpha [TNF-α], and interleukin [IL]-2, IL-4, and IL-6), fear of movement, spinal range of motion (ROM), and disability level.
- Study design: RCT.

No restrictions were imposed on language or publication date.

### Study Selection

The retrieved studies were imported into Endnote X9 software (Clarivate), which was used to eliminate duplicate studies. Two independent reviewers (authors TTZ and FW) performed the initial screening of the literature by reading the titles and abstracts of all retrieved studies, and studies that did not meet the inclusion criteria were excluded. Next, the full texts of the remaining studies were screened. Any disagreements were resolved by negotiation and discussion with a third reviewer (author XZ).

### Data Extraction

Two independent reviewers (authors FW and XL) extracted detailed information, including the name of the first author, the year and country of publication, the language of publication, study design, the number of included subjects (% female), diagnosis, and outcome indicators. Information about the characteristics of the interventions, including dose, frequency, and duration, was also collected for both the VR group and the control group. The sample size and mean (SD) of the outcome indicators in each group were collected. When the same group of participants was reported in different studies, the group with the largest sample size was selected for inclusion in this review to avoid duplicate publications [28]. For information that could not be confirmed, the authors were contacted by email. The 2 reviewers cross-checked the data at the end of the extraction, and any disagreements were resolved by negotiation.

### Risk-of-Bias Assessment

The methodological quality of the included studies was independently assessed by 2 reviewers (authors XL and ZFH) using the Cochrane Risk of Bias tool, and the studies were classified as having a low, unclear, or high risk of bias [29]. Disagreements were resolved by consulting a third reviewer (author QD). The Egger test and funnel plots generated with Stata 14.0 software (StataCorp) were used to evaluate potential publication bias. The trim-and-fill method was used to adjust for funnel plot asymmetry due to publication bias [30]. Sensitivity analyses were performed by removing each study separately to assess the robustness of the results [29]. The overall strength of the evidence was assessed using the Grading of Recommendations, Assessment, Development and Evaluation (GRADE) criteria [31].

### Meta-Analysis and Subgroup Analysis

This systematic review and meta-analysis were performed using Review Manager software 5.4 (Informer Technologies) and Stata 14.0 software. Heterogeneity was tested using the *I*^2^ statistic. A fixed effects model was selected for the outcome indicators if *I*^2^<50%, while a random effects model was used when there was significant statistical heterogeneity (*I*^2^>50%, *P*<.05). The effect size used to synthesize the outcome measure was the weighted mean difference (WMD). Three subgroup analyses were performed to explore the possible causes of heterogeneity among the studies: the region of CSP (CNP vs CLBP), VR types (IVR vs NIVR), and treatment duration (<4 weeks vs ≥4 weeks).

## Results

### Search Results

**Figure 1:**
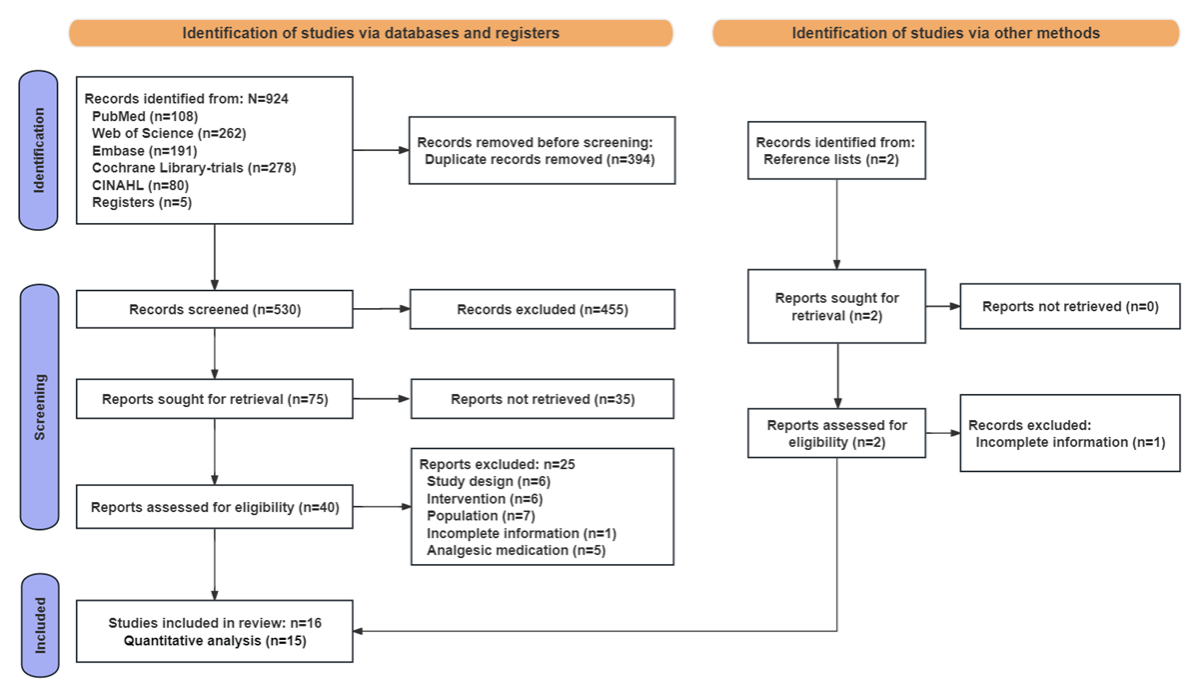
PRISMA flowchart: database and clinical trial register search and other sources. PRISMA: Preferred Reporting Items for Systematic Reviews and Meta-Analysis.

A total of 924 records were obtained from the 5 databases and the trial registration platform. A total of 394 (42.6%) duplicates were identified and removed using Endnote X9 software. After screening the titles and abstracts, 40 (7.5%) of the remaining 530 RCTs were retained, and 490 (92.5%) were excluded for the following reasons: (1) the study population included patients without CSP, (2) the intervention did not use VR therapy, (3) the type of study was a non-RCT, (4) the information was incomplete, and (5) the patients also received analgesic medication. Of the 40 studies, 15 (38%) were retained after reading the full text and 25 (62%) were excluded for the following reasons: (1) the study population included patients without CSP, (2) the intervention did not use VR therapy, (3) the type of study was a non-RCT, (4) the information was incomplete, and (5) the patients also received analgesic medication. Two additional RCTs were retrieved from the reference lists of the included studies. One RCT was retained after the full text was read, and the other was excluded due to incomplete information. A total of 16 studies were included in this review, 15 (94%) of which reported sufficient data (eg, mean [SD], sample size) on the analgesic effect of VR for CSP. Therefore, 15 studies were included in the meta-analysis. The PRISMA flowchart of selecting the included studies is shown in Figure 1.

The CSP reported in the included studies included CLBP [32-43] and CNP [44-47]. All patients had chronic pain that persisted for more than 3 months. The sample size varied from 8 to 90 participants, and the mean age ranged from 18 to 85 years. The characteristics of all the studies are summarized in Table 1.

**Table 1:** Characteristics of the included studies [32-47].

First author	Patient characteristics				Outcome measures		Time points		Dropout rate (%)		Country, language
	Participants, n (% female)	Age (years), mean (SD)	Diagnosis								
Garcia et al [32]	T^a^: 179 I^b^: 89 (75) C^c^: 90 (78)	I: 51.5 (13.5) C: 51.4 (12.9)	CLBP^d^	DVPRS^e^, Pain Catastrophizing Scale (PCS), 8-item Chronic Pain Acceptance Questionnaire (CPAQ-8)		Baseline, –7, 0, 4, 7, 11, 14, 18, 21, 25, 28, 32, 35, 39, 42, 46, 49, 53, 56 days		I: 0 C: 0		United States, English	
Nambi et al [33]	T: 60 I (VR^f^): 20 I (core stabilization [CS]): 20 C: 20	I (VR): 21.45 (1.50) I (CS): 21.39 (1.40) C: 20.97 (1.50)	CLBP	NPRS^g^, quality of life (physical fitness index)		Baseline, 4 weeks, 8 weeks, 6 months		I (VR): 0.05 I (CS): 0.05 C: 0		Saudi Arabia, English	
Nambi et al [34]	T: 45 I (VR): 15 I (isokinetic training [IKT]): 15 C: 15	I (VR): 20.23 (1.60) I (IKT): 21.25 (1.20) C: 20.78 (1.60)	CLBP	NPRS		Baseline, 4 weeks		I (VR): 0 I (IKT): 0 C: 0		Saudi Arabia, English	
Yalfani et al [35]	T: 25 I: 13 C: 12	I: 68.00 (2.94) C: 67.08 (2.90)	CLBP	VAS^h^, 36-item Short Form Health Survey (SF-36)		Baseline, 8 weeks		I: 0 C: 0		Iran, English	
Park et al [36]	T: 24 I (NWE^i^): 8 I (lumbar stabilization exercise [LSE]): 8 C: 8	I (NWE): 44.12 (5.48) I (LSE): 43.37 (5.42) C: 45.50 (5.34)	CLBP	VAS		Baseline, 8 weeks		I: 0 C: 0		South Korea, English	
Afzal et al [37]	T: 90 I: 45 (64.28) C: 45 (69.04)	I: 37.5 (12.5) C: 38.2 (11.8)	CLBP	VAS, Modified Oswestry Disability Index		Baseline, 4th, 8th 12th sessions		I: 0.07 C: 0.07		Pakistan, English	
Nambi et al [38]	T: 60 I (VRE^j^): 20 I (isokinetic exercise [IKE]): 20 C: 20	I (VRE): 23.2 (1.6) I (IKE): 22.9 (1.7) C: 22.8 (1.8)	CLBP	VAS, inflammatory biomarkers		Baseline, 4 weeks		I (VRE): 5 I (IKE): 5 C: 0		Saudi Arabia, English	
Nambi et al [39]	T: 36 I (VR): 12 I (combined physical rehabilitation [CPR]): 12 C: 12	I (VR): 21.3 (2.6) I (CPR): 21.8 (2.2) C: 20.9 (2.8)	CLBP	Inflammatory biomarkers		Baseline, 4 weeks		I (VR): 0 I (CPR): 0 C: 0		Saudi Arabia, English	
Nambi et al [40]	T: 54 I (VR): 18 I (CPR): 18 C: 18	I (VR): 22.3 (1.6) I (CPR): 21.4 (1.8) C: 21.9 (1.8)	CLBP	VAS, TSK-17		Baseline, 4 weeks		I (VR): 0 I (CPR): 0 C: 0		Saudi Arabia, English	
Matheve et al [41]	T: 84 I: 42 (64) C: 42 (64)	I: 42.1 (11.5) C: 44.2 (11.9)	CLBP	NPRS, Roland-Morris Disability Questionnaire (RMDQ), PCS		Baseline, postintervention		I: 0 C: 0		Belgium, English	
Stamm et al [42]	T: 22 I: 11 (73) C: 11 (55)	I: 75.00 (5.80) C: 75.50 (4.39)	CLBP	NRS^k^, Chronic Pain Grade Questionnaire (CPGQ), 12-item Short Form Health Survey (SF-12), Hannover Functional Ability Questionnaire for Measuring Back Pain–Related Disability (Ffb-H-R), TSK^l^-11		Baseline, 4 weeks		I: 0 C: 0		Germany, English	
Monteiro-Junior et al [43]	T: 34 I: 17 (100) C: 17 (100)	T: 68 (4)	CLBP	NRS		Baseline, 8 weeks		I: 17.6 C: 5.8		Brazil, English	
Cetin et al [44]	T: 41 I: 21 C: 20	I: 40.00 (11.88) C: 41.94 (10.76)	CNP^m^	Joint position sense error (JPSE), VAS, pressure pain threshold (PPT), SF-36		Baseline, 6 weeks		I: 19 C: 15		Turkey, English	
Bahat et al [45]	T: 90 I (VR): 30 (63) I (laser): 30 (70) C: 30 (77)	I (VR): 48.00 (14.07) I (laser): 48.00 (17.41) C: 48.00 (17.76)	CNP	NDI^n^, VAS, EQ-5D, TSK-17, cervical range of motion (CROM), kinematic measures		Baseline, 4 weeks		I (VR): 16.6 I (laser): 13.3 C: 16.6		Israel, English	
Nusser et al [46]	T: 55 I (VR): 17 (53) I (sensorimotor group [SM]): 16 (69) C: 18 (66)	I (VR): 51.2 (8.8) I (SM): 53.1 (5.7) C: 49.8 (8.1)	CNP	NRS, active cervical range of motion (ACROM), NDI		Baseline, 3 weeks		I (VR): 0 I (SM): 11 C: 10		Germany, English	
Tejera et al [47]	T: 44 I: 22 (50) C: 22 (54.5)	I: 32.72 (11.63) C: 26.68 (9.21)	CNP	VAS, conditioned pain modulation (PPT), ACROM device, NDI, PCS, 11-item Spanish version of the TSK		Baseline, 4 weeks, 1 month, 3 months		I: 0 C: 0		Spain, English	

^a^T: total participants.

^b^I: intervention group.

^c^C: control group.

^d^CLBP: chronic low back pain.

^e^DVPRS: Defense and Veterans Pain Rating Scale.

^f^VR: virtual reality.

^g^NPRS: Numerical Pain Rating Scale.

^h^VAS: Visual Analogue Scale.

^i^NWE: Nintendo Wii exercise.

^j^VRE: virtual reality exercise.

^k^NRS: Numeric Rating Scale.

^l^TSK: Tampa Scale for Kinesiophobia.

^m^CNP: chronic neck pain.

^n^NDI: Neck Disability Index.

The types of VR interventions included IVR [32,35,42,44-47] and NIVR [33,34,36-41,43], which were classified based on the degree of isolation participants experienced when interacting with the virtual environment during VR therapy. NIVR uses a wall-mounted screen or a computer monitor as the vehicle for VR content, while IVR uses a headset or head-mounted display [48]. Compared to NIVR, IVR can increase the user’s sense of presence by improving immersion through the addition of auditory or haptic feedback [49]. The duration of a single VR session ranged from 2 to 40 minutes, and the frequency of treatment ranged from 5 to 7 times a week; all the included studies ranged in duration from a single exercise session to 8 weeks. For the control groups, 5 (31%) studies performed conventional balance function training [33,34,38-40], 5 (31%) performed conventional physical therapy [36,37,41,46,47], 2 (13%) performed core training [43,44], and the remaining conducted treatments, including sham VR [32], conventional multimodal pain therapy [42], waiting lists [45], and standard care [35]. The intervention details are summarized in Table 2.

**Table 2:** Characteristics of the intervention protocols used in the included studies [32-47].

First author	Intervention group	Control group	Device	Duration
Garcia et al [32]	Ease VR^a^, IVR^b^, interactive, pain education, relaxation/interception, mindful escape, pain distraction games, dynamic breathing performed 56 times (2-16 minutes each time, average of 6 minutes, 1 time/day)	Sham VR, NIVR^c^, not interactive, displayed 2D nature footage with neutral music, 20 videos rotated over 56 sessions, performed 56 times (2-16 minutes each time, average of 6 minutes, 1 time/day)	Pico G2 4K all-in-one head-mounted VR device	8 weeks
Nambi et al [33]	VR group: sit in the virtual platform and select firing game executed by trunk movements (flexion, extension, and lateral flexion; 30 minutes/day, 5 times/week, for 4 weeks); heat modality (20 minutes); therapeutic ultrasound (25 minutes)	Conventional balance function training, traditional active balance exercise for abdominal and back muscles (5 times/week for 4 weeks); heat modality (20 minutes); therapeutic ultrasound (25 minutes)	VR group: Pro-Kin system PK 252 N (TecnoBody)	4 weeks
Nambi et al [34]	VRT: shooting game (30 minutes, 5 days/week, for 4 weeks); home-based exercise; hot-pack therapy (20 minutes); ultrasound (frequency 1 MHz, intensity 1.5 W/cm^2^ in continuous form for 5 minutes)	Conventional balance function training: standardized conventional exercises actively involving abdominal, deep abdominal, and back muscles (30 minutes/session, 5 days/week, for 4 weeks); hydrocollator packs (20 minutes/session); continuous ultrasound (frequency 1 MHz, intensity 1.5 W/cm^2^) at the low back region (5 minutes, 5 days/week, for 4 weeks)	VRT: Pro-Kin system (TecnoBody)	4 weeks
Yalfani et al [35]	Fishing, boxing, tennis, football, bowling, beat saber, audio shield, and skiing (30 minutes, 3 times/week, for 8 weeks)	Standard care.	VR: HTC Vive virtual reality system	8 weeks
Park et al [36]	NWE^d^: using the Nintendo Wii exercise program, including the wakeboard, Frisbee dog, jet ski, and canoe games. Participants chose which Nintendo Wii sports program to perform and took a 2-minute break every 10 minutes (30 minutes/session, 3 times/week, for 8 weeks)	Conventional physical therapy: using physical agent modalities, such as a hot pack (30 minutes); interferential current therapy (15 minutes); deep heat with ultrasound (5 minutes)	VR: Nintendo	8 weeks
Afzal et al [37]	Kinetic exergames (trunk slide flexion, sitting to avoid obstacles, jumping and combined movement of arms, for 5 minutes); after 30 seconds of rest, play body ball game for 5 minutes (3 sessions/week for a total of 12 sessions); routine physical therapy	Conventional physical therapy: heat therapy for 10 minutes, hamstring stretching, back-strengthening exercises (3 sessions/week for a total of 12 sessions)	VR: nonimmersive system with a kinetic device (model V.2), incorporated with red-green-blue (RGB) cameras and time-of-flight (TOF) sensor, attached with a liquid crystal display (LCD) screen	4 weeks
Nambi et al [38]	VRE^e^: virtual training exercises performed in the upright position, a car race game chosen from the list of games, and training given to focus on the back muscles. The participant was asked to sit on the moving game chair and instructed to watch the game on the desktop monitor (30 minutes/session, 5 days/week, for 4 weeks); hydrocollator packs (20 minutes/session); continuous ultrasound (frequency 1 MHz, intensity 1.5 W/cm^2^) at the low back region (5 minutes, 5 days/week, for 4 weeks)	Conventional balance function training: standardized conventional exercises actively involving abdominal, deep abdominal, and back muscles (30 minutes/session, 5 days/week, for 4 weeks); hydrocollator packs (20 minutes/session); continuous ultrasound (frequency 1 MHz, intensity 1.5 W/cm^2^) at the low back region (5 minutes, 5 days/week, for 4 weeks)	VRE: Pro-Kin system (TecnoBody)	4 weeks
Nambi et al [39]	Virtual reality training (VRT): shooting game, sitting on a virtual platform and visualizing the game on the computer display screen (30 minutes each time, 5 times/week, for 4 weeks); heat modality (20 minutes); therapeutic ultrasound (frequency 1 MHz, intensity 1.5 W/cm^2^; 5 minutes); home-based exercise (10 repetitions, bottom-to-heel stretch, opposite arm/leg raise, back extension, bridging, knee rolling; 2 times/day for 4 weeks)	Conventional balance function training: active isotonic and isometric exercises for abdominal, deep abdominal, and back muscles (10-15 repetitions/day, 5 days/week for 4 weeks; stretching focused on each muscle group for 3 repetitions for 10 seconds per muscle group); heat modality (20 minutes); therapeutic ultrasound (frequency 1 MHz, intensity 1.5 W/cm^2^; 5 minutes); home-based exercise (10 repetitions, bottom-to-heel stretch, opposite arm/leg raise, back extension, bridging, knee rolling; 2 times/day for 4 weeks)	VR: Pro-Kin system PK 252 N (Pelvic Module balance trunk MF; TecnoBody)	4 weeks
Nambi et al [40]	VRT: shooting game, sitting on a virtual platform and visualizing the game on the computer display screen (30 minutes each time, 5 times/week, for 4 weeks); heat modality (20 minutes); therapeutic ultrasound (frequency 1 MHz, intensity 1.5 W/cm^2^; 5 minutes)	Conventional balance function training: active isotonic and isometric exercises for abdominal, deep abdominal, and back muscles (10-15 repetitions/day, 5 days/week for 4 weeks; stretching focused on each muscle group for 3 repetitions for 10 seconds per muscle group); heat modality (20 minutes); therapeutic ultrasound (frequency 1 MHz, intensity 1.5 W/cm^2^; 5 minutes)	VR: Pro-Kin system PK 252 N (Pelvic Module balance trunk MF; TecnoBody)	4 weeks
Matheve et al [41]	2 different games (2 minutes each); single-session intervention, 2 × 2 minutes of pelvic tilt exercises in the sagittal plane, with 30 seconds of rest in between, through a wireless motion sensor	Conventional physical therapy: 2 different games (2 minutes each); single-session intervention, 2 × 2 minutes of pelvic tilt exercises in the sagittal plane, with 30 seconds of rest in between	VR: wireless motion sensor (Valedo Pro, Hocoma)	Single exercise session
Stamm et al [42]	Multimodal pain therapy in VR (movement therapy and psychoeducation), training session including 12 exercises, structured as follows: (1) warm-up (training of upper and lower extremities), (2) main part (strengthening of abdominal and back muscles, core stability), (3) cool-down (stretching, progressive muscle relaxation), (4) psychoeducative units (topics: physiology of pain, pain management, stress management, everyday training), 3 times/week for 30 minutes	Conventional multimodal pain therapy: chair-based group exercises and psychoeducation in a group setting), 3 times/week for 30 minutes	VR: head-mounted display headset using the ViRST VR app	4 weeks
Monteiro-Junior et al [43]	Virtual physical training (8 exercises, 30 minutes each time, with 3 weekly sessions lasting 90 minutes each), lasted 8 weeks, 3 times weekly/session	Core training: postures adopted by participants for 15-30 seconds or according to the capacity of each; 10-15 seconds between postures (ie, bridges), with each performed 3 times, lasted 8 weeks, 3 times weekly/session	VR: Wii Balance Board (WBB; Nintendo)	8 weeks
Cetin et al [44]	VR exercises: VR apps that allowed neck movements in all directions, motor control (MC) exercises (20 minutes and then VR for 20 minutes, 5 repetitions for each exercise; 40 minutes/session, 3 sessions/week, for 6 weeks, total of 18 sessions)	Core training: strengthening of deep cervical flexors (DCFs), deep cervical extensors (DCEs), and axioscapular muscles; stretching exercises; and postural correction exercises (40 minutes, 10 repetitions for each exercise, 3 sessions/week, for 6 weeks, total of 18 sessions)	VR: Oculus Go VR glasses, 2 VR apps installed: “Ocean Rift” and “Gala 360”	6 weeks
Bahat et al [45]	VR group: kinematic home training and customized software with the virtual airplane controlled by head motion (5 minutes, 4 times/day, 20 minutes/day, 4 times/week, for 4 weeks)	Waiting list	VR: customized neck VR system (hardware including Oculus Rift DK1 head-mounted display equipped with 3D motion tracking; software developed using Unity-pro, version 3.5, Unity Technologies)	4 weeks
Nusser et al [46]	VR group: neck-specific sensorimotor training (NSST)— head-repositioning test (HRT), head-to-target test (HTT), dynamic exercise including 5 different trajectories (3 minutes given between tasks), training divided into 6 20­minute sessions for a total of 120 minutes); standard rehabilitation program	Conventional physical therapy: different forms of general and neck-specific exercise therapies (strengthening, mobilization, relaxation, medical training therapy, functional gymnastics, aqua therapy, physical therapy, and traditional “back school”)	VR: modified VR system (Fraunhofer Institute für Graphische Datenverarbeitung), helmet (Schutz helm uvex pheos alpine, Fürth), 3Space Fastrak System (Polhemus Inc)	3 weeks
Tejera et al [47]	VR mobile apps “Full Dive VR,” only lateral flexion movements of the neck; “VR Ocean Aquarium 3D”: flexion, extension, and rotation movements (3 series of 10 repetitions, with 30 seconds of rest between exercises)	Conventional physical therapy: flexion, extension, rotation, and tilt exercises (3 series of 10 repetitions, with 30 seconds of rest between exercises)	VR: VR Vox Play glasses with a head-mounted display clamping system (weight 330 g) with an LG Q6 smartphone attached, 2 VR mobile apps installed	4 weeks

^a^VR: virtual reality.

^b^IVR: immersive virtual reality.

^c^NIVR: nonimmersive virtual reality.

^d^NWE: Nintendo Wii exercise.

^e^VRE: virtual reality exercise.

**Figure 2:**
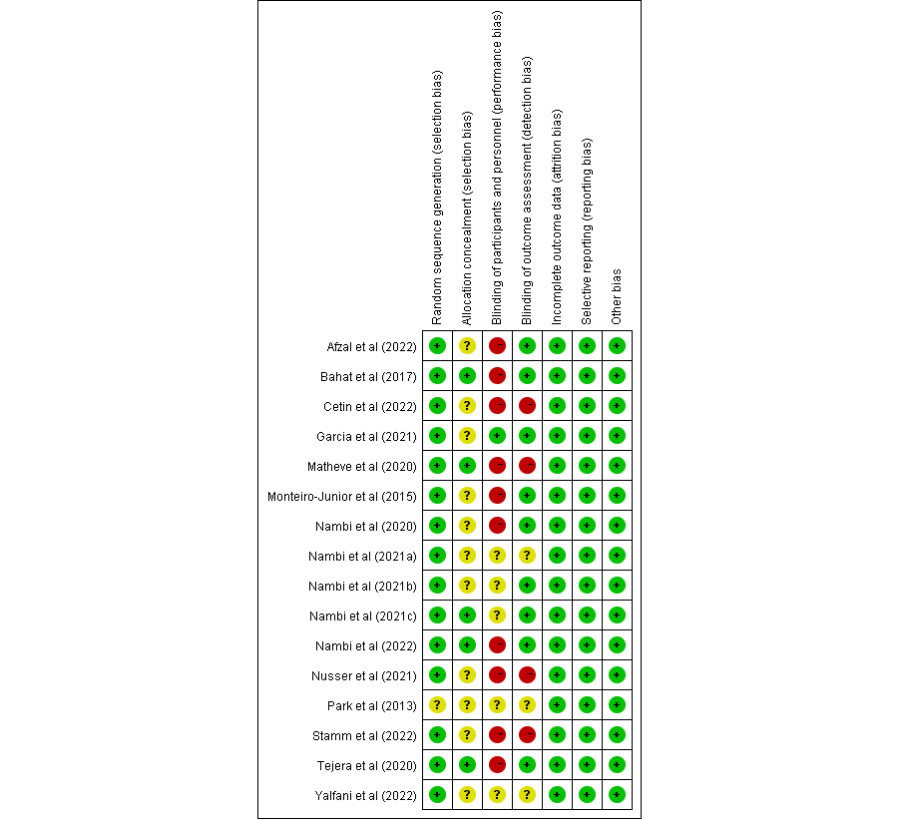
Cochrane risk-of-bias summary for included studies.

The risk of bias in the 16 (100%) studies included in the meta-analysis is presented in Figure 2. Overall, 10 (63%) studies showed a high risk of bias. In addition, 15 (94%) RCTs generated an adequately randomized sequence, and 9 (60%) of them were analyzed using a blinded method for outcome measurement. Ratings using the GRADE methodology for all outcome measurements were inconsistent and ranged from moderate to low quality (Multimedia Appendix 3). Therefore, the quality of evidence from most studies was classified as fair.

### Primary Outcome

#### Pain Intensity

All 16 (100%) studies (800 patients) reported pain intensity: 9 (56%) used the Visual Analogue Scale (VAS) [34-38,40,44,45,47], 2 (13%) used the Numerical Pain Rating Scale (NPRS) [33,41], 3 (19%) used the Numeric Rating Scale (NRS) [42,43,46], and 1 (6%) used the Defense and Veterans Pain Rating Scale (DVPRS) [32]. The random effects model revealed that compared with the control treatment, the VR intervention significantly reduced pain intensity (WMD=–1.63, 95% CI –2.11 to –1.16, *P*<.001, *I*^2^=90%). Clinical differences between groups were significant, and as suggested, the minimal clinically important difference (MCID) threshold on the VAS for LBP was set at a 1.5-point reduction [50]. Given the significant heterogeneity observed (*I*^2^=90%), we performed subgroup analyses to investigate the source of heterogeneity based on the different regions, VR types, and treatment durations.

**Figure 3:**
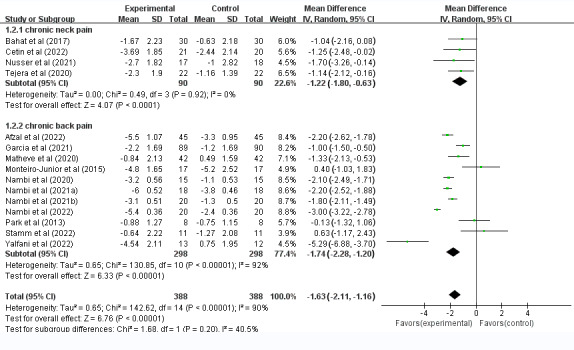
Forest plots of the effect of VR compared with other treatments on pain intensity in patients with CSP: subgroup analysis of posttreatment effectiveness for different regions of spinal pain. CSP: chronic spinal pain; VR: virtual reality.

**Figure 4:**
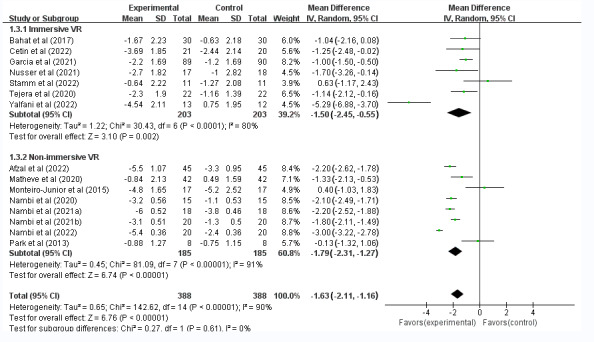
Forest plots of the effect of VR compared with other treatments on pain intensity in patients with CSP: subgroup analysis of posttreatment effectiveness for the VR intervention type. CSP: chronic spinal pain; VR: virtual reality.

VR had a good analgesic effect on both CNP and CLBP groups compared with the control group. The results did not significantly differ among the subgroups (WMD=−1.63, 95% CI −2.11 to −1.16); see Figure 3. Moreover, a total of 7 (44%) studies demonstrated that IVR significantly improved CSP (WMD=–1.50, 95% CI –2.45 to –0.55, *P*<.001, *I*^2^=80%) [32,35,42,44-47]. Another 8 (50%) studies showed that NIVR improved CSP substantially (WMD=–1.50, 95% CI –2.45 to –0.55, *P*<.001, *I*^2^=90%) [33,34,36-38,40,41,43]; see Figure 4. The subgroup analyses also revealed significant differences between treatment durations of <4 weeks (WMD=–1.41, 95% CI –2.12 to –0.69, *P*=.001, *I*^2^=0%) and ≥4 weeks (WMD=–1.65, 95% CI –2.16 to –1.14, *P*<.001, *I*^2^=91%) in terms of the analgesic effect of VR treatment on CSP (Multimedia Appendix 4).

### Secondary Outcomes

#### Inflammatory Markers

Patients with CSP develop a systemic inflammatory response and have elevated levels of inflammatory markers in the blood [51]. Two studies (62 patients) focused on the levels of inflammatory markers (eg, CRP, TNF-α, IL-2, IL-4, and IL-6) by collecting 10 mL of venous blood [38,39]. The results showed that VR therapy significantly improved the level of CRP (WMD=–0.89, 95% CI –1.07 to –0.70, *P*<.001, *I*^2^=0%), TNF-α (WMD=–6.60, 95% CI –8.56 to –4.64, *P*<.001, *I*^2^=98%), and IL-6 (WMD=–2.76, 95% CI 2.98 to –2.53, *P*<.001, *I*^2^=0%). No significant differences were found between the IL-2 and IL-4 subgroups (Figure S1 in Multimedia Appendix 5).

#### Fear of Movement

Four studies (162 patients) reported fear of movement according to the 11-item or 17-item Tampa Scale of Kinesiophobia (TSK-11 or TSK-17, respectively) [42,47]. No significant differences were found in either the TSK-11 (WMD=–0.81, 95% CI –4.48 to 2.86, *P*=.66, *I*^2^=0%; Figure S2 in Multimedia Appendix 5) or TSK-17 (WMD=–9.66, 95% CI –22.01 to 2.68, *P*=.13, *I*^2^=97%; Figure S3 in Multimedia Appendix 5).

#### Spinal Range of Motion

Three studies reported changes in the ROM of the neck in 4 directions before and after the intervention [45-47]. No significant differences were found between the groups in terms of flexion (WMD=2.67, 95% CI –2.31 to 7.64, *P*=.29, *I*^2^=61%), extension (WMD=3.92, 95% CI –2.17 to 10.0, *P*=.21, *I*^2^=48%), right rotation (WMD=–0.22, 95% CI –4.38 to 3.95, *P*=.92, *I*^2^=0%), or left rotation (WMD=0.08, 95% CI –3.90 to 4.05, *P*=.97, *I*^2^=42%); see Figure S4 in Multimedia Appendix 5.

#### Disability Level

Three studies (139 patients) reported disability levels in patients with CNP by using the Neck Disability Index (NDI) [45-47], a 10-item questionnaire that assesses self-reported disability related to CNP. Higher scores on the NDI indicate higher levels of disability. No significant differences were found in the pooled analysis of 3 (19%) studies (WMD=–2.66, 95% CI –5.47 to 0.15, *P*=.06, *I*^2^=48%); see Figure S5 in Multimedia Appendix 5.

#### Adverse Events

One study reported that after 1 month of intervention, patients experienced nausea and motion sickness [32], two studies reported that there were no adverse events [33,37], and the remaining studies did not mention adverse events. The overall dropout rate was 4.25% (17/400) in the intervention group and 3.75% (15/400) in the control group.

### Publication Bias and Sensitivity Analysis

**Figure 5:**
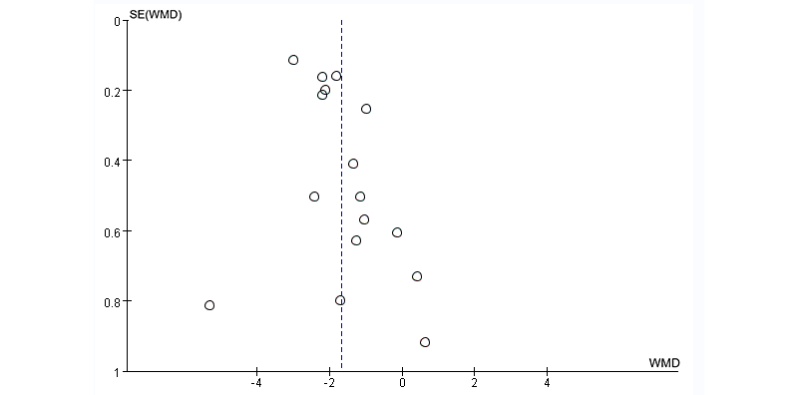
Funnel plot of pain intensity in the VR group compared with the control group. VR: virtual reality; WMD: weighted mean difference.

**Figure 6:**
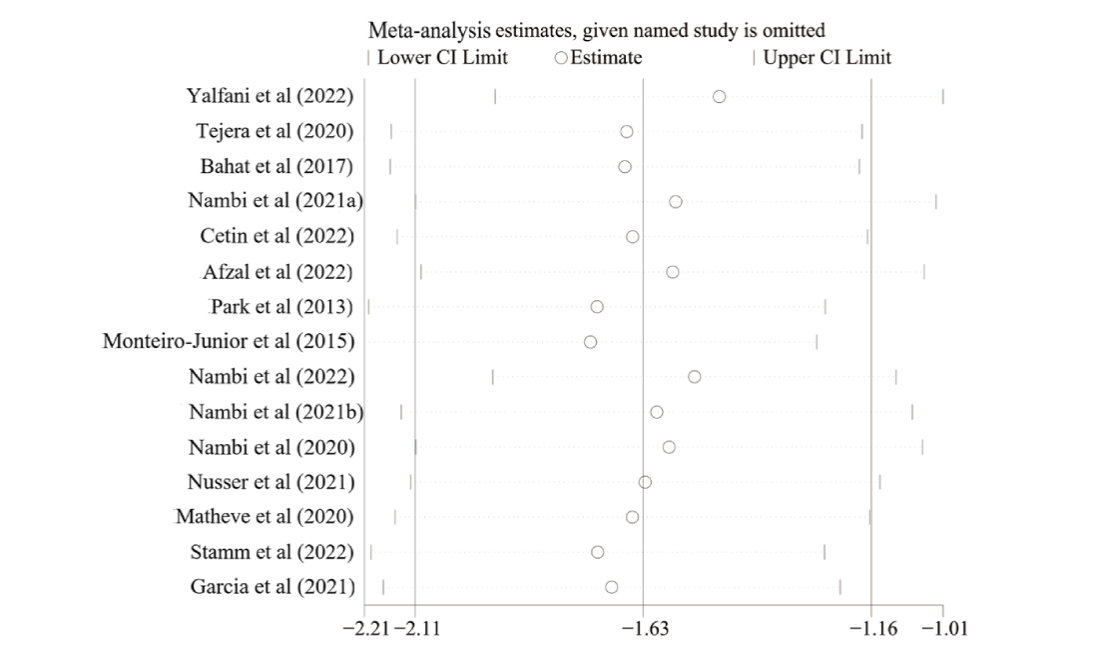
Sensitivity analysis of the included studies.

**Figure 7:**
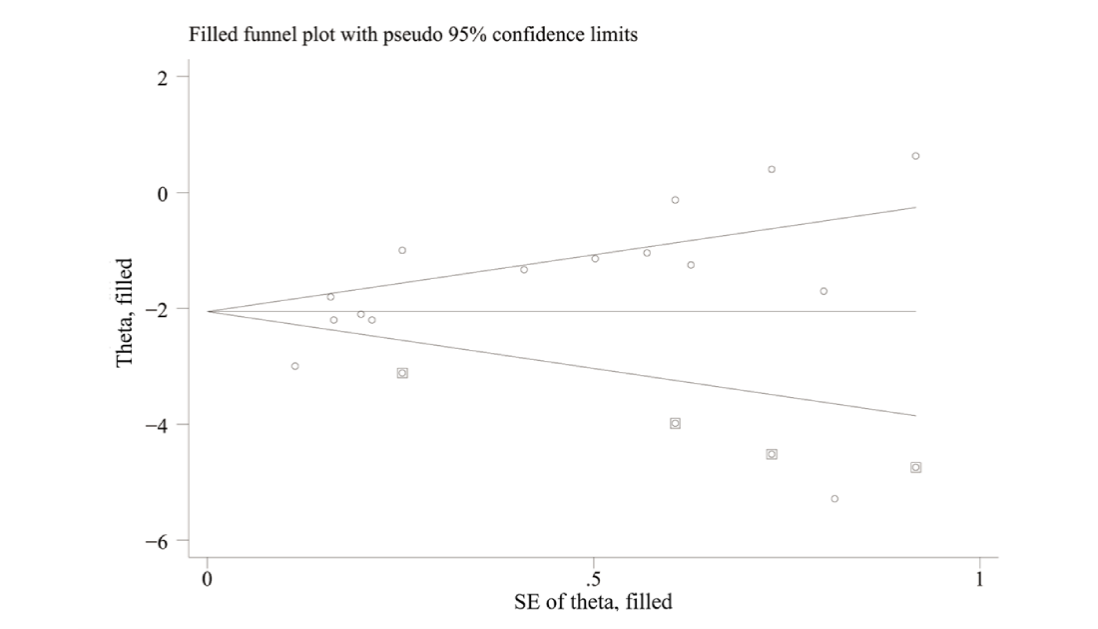
Trim-and-fill analysis to estimate the number of potentially missing studies about the effect of VR on CSP. Circles represent real studies, and squares represent studies estimated by this method. Circles represent real studies, and squares represent studies estimated by this method. CSP: chronic spinal pain; VR: virtual reality.

The Egger test indicated significant publication bias in the results for pain intensity (*P*=.03; Figure 5). The sensitivity analysis for pain intensity revealed that removing each study separately did not significantly affect the pooled results, thus indicating that the results are robust (Figure 6). The trim-and-fill method was performed, and it was estimated that there were 4 missing studies. The pooled estimates (95% CIs) calculated for the fixed effects model and the random effects model were –2.30 (–2.42 to –2.18) and –2.06 (–2.50 to –1.61), respectively (Figure 7). No significant changes in the results were observed before or after pruning or filling, indicating that our results are robust and plausible.

## Discussion

### Principal Findings

The primary purpose of this meta-analysis was to compare the relative efficacy of VR therapy and other therapies (eg, conventional therapy, sham stimulation, and standard care) for treating CSP. The results indicated that VR therapy can effectively relieve CSP. The results of subgroup analyses showed that VR is a beneficial pain management strategy for patients with CNP and CLBP. For different types of VR, subgroup analyses showed that compared to the control group, IVR and NIVR both significantly improved CSP. No statistically significant differences were found between patients who underwent VR treatments for a duration of <4 weeks and a duration of ≥4 weeks. VR was associated with a significant improvement in inflammatory marker levels but not in the fear of movement, spinal ROM, or disability level. VR was found to be well tolerated among these patients.

### Discussion of the Results

The primary result suggested that VR reduces self-reported pain intensity, which might be explained by several implicating mechanisms [52,53]. A previous study reported that abdominal muscle strength is significantly lower in people with LBP [54], and a lack of strength in the core trunk muscles can lead to a decrease in intra-abdominal pressure, affecting spinal stability [55]. VR, as a novel human-computer interaction approach, can stimulate and mobilize the sensory system during training and results in changes in neuroplasticity and enhanced performance of relevant muscle groups, promoting a new motor learning process and leading to increased spinal stability [37,56], which would benefit pain relief. Furthermore, previous studies have reported that an intervertebral disc undergoes aging or pathological changes in the adjacent region in patients with CSP, exposing cells within the nucleus pulposus to macrophages, resulting in an inflammatory response that might trigger pain [7,8]. VR therapy may enhance the activity of disc fibroblasts and increase the thickness of the multifidus muscle [39,57], which is beneficial for relieving pain intensity. Furthermore, pain is an unpleasant subjective sensation associated with actual or potential tissue damage and is correlated with the degree of patient attention given to the pain area [58-61]. The various virtual game environments and real-time feedback methods are the most eye-catching features in the VR training process; these methods can be used to attract the patient’s visual and auditory attention to achieve motor performance, while relatively less attention has been given to the effects of VR on pain [62,63].

Although the high heterogeneity of the primary outcome and the results of the subsequent subgroup analyses suggest that the region of CSP, VR type, and treatment duration may play a role in the heterogeneity, the results of the sensitivity analysis indicate that these differences are more likely to be caused by 6 studies [33-35,37,38,40], which included participants of different ages.

VR therapy significantly improved the levels of inflammatory markers, including CRP, TNF-α, and IL-6. Numerous studies have previously reported an association between CSP and changes in inflammatory cytokines, such as IL-1 and TNF-α, which are thought to be closely related to the pathogenesis of disc herniation and degeneration [64,65]. Similarly, Nambi et al [66] reported that 4 weeks of VR training could significantly decrease pain intensity, increase functional impairment, and improve CRP, TNF-α, IL-2, IL-4, and IL-6 levels. However, the limited number and low quality of the included studies need to be noted, and further RCTs with large samples and rigorous study designs are needed to elucidate these results.

Patients with CSP may engage in fear/avoidance behaviors to avoid pain and protect themselves by limiting spinal motion, which ultimately affects spinal mobility and the speed of movement [67,68], with the degree of pain catastrophizing being proportional to the degree of disability [69,70]. However, we found no statistically significant differences in fear avoidance beliefs after the VR intervention but at the 3-month follow-up [47]. A systematic review and meta-analysis reported that VR therapy enhances spinal ROM and physical functioning in patients with CNP [26]. We failed to observe significant differences in the spinal ROM or disability level after VR intervention compared to those in the control group, which may be attributed to the relatively short duration (0-8 weeks) of the VR intervention (the reported mean duration was 4.81 weeks).

### Limitations

Several limitations need to be addressed in this meta-analysis. First, the pooled analysis of the studies may be imprecise due to the large heterogeneity and the low quality of evidence from most of the included studies, and the results should be interpreted with caution. Second, the optimal duration of treatment for CSP could not be determined. Third, the effectiveness of VR therapy in patients with CSP and its analgesic effects in long-term follow-up must be further explored in high-quality studies. Fourth, indicators related to quality of life, such as depression and anxiety, should be emphasized and investigated in depth in future studies of patients with CSP.

### Conclusion

VR therapy is an innovative and effective analgesic method that has beneficial effects on inflammatory markers in patients with CSP compared to other therapies (sham stimulation, usual care, conventional treatment). However, this approach may not have significant effects on the fear of movement, spinal ROM, or disability level. Notably, the quality of the evidence from the RCTs included in this study ranged from moderate to low. Therefore, we recommend that readers interpret the results of this study with caution. Future trials with large sample sizes, rigorous designs, and long-term follow-up periods are needed to explore the clinical significance of these differences and key issues in patients with CSP and to elucidate the underlying mechanisms of VR.

## Appendix

Multimedia Appendix 1 The PRISMA checklist. PRISMA: Preferred Reporting Items for Systematic Reviews and Meta-Analysis.

Multimedia Appendix 2 Search strategy for all electronic databases.

Multimedia Appendix 3 The GRADE criteria. GRADE: Grading of Recommendations, Assessment, Development and Evaluation.

Multimedia Appendix 4 Forest plots of the effect of VR compared with other treatments for pain intensity in patients with CSP: subgroup analysis of posttreatment effectiveness for treatment duration. CSP: chronic spinal pain; VR: virtual reality.

Multimedia Appendix 5 Forest plots of the effect of VR compared with other treatments for inflammatory marker level, fear of movement, spinal ROM, and disability level in patients with CSP. CSP: chronic spinal pain; ROM: range of motion; VR: virtual reality.

## Abbreviations

CLBP: chronic low back pain
CNP: chronic neck pain
CRP: C-reactive protein
CSP: chronic spinal pain
DVPRS: Defense and Veterans Pain Rating Scale
GRADE: Grading of Recommendations, Assessment, Development and Evaluation
IL: interleukin
IVR: immersive virtual reality
LBP: low back pain
NDI: Neck Disability Index
NIVR: nonimmersive virtual reality
NPRS: Numerical Pain Rating Scale
NRS: Numeric Rating Scale
NSAID: nonsteroidal anti-inflammatory drug
PRISMA: Preferred Reporting Items for Systematic Reviews and Meta-Analysis
RCT: randomized controlled trial
ROM: range of motion
TNF-α: tumor necrosis factor-alpha
TSK: Tampa Scale of Kinesiophobia
VAS: Visual Analogue Scale
VR: virtual reality
VRE: virtual reality exercise
WMD: weighted mean difference

## Data Availability

All data generated or analyzed during this study are included in this published paper and its supplementary information files.
