# Supplementary material for: Virtual Reality Therapy for the Management of Chronic Spinal Pain: Systematic Review and Meta-Analysis
Source: JMIR Serious Games. 2024 Feb 12;12:e50089. doi: 10.2196/50089 (PMC10897798; doi:10.2196/50089)
Supplement: Multimedia Appendix 2 [file games_v12i1e50089_app2.docx]

2. Search Strategies

**PUBMED (108) 2023.11.11**

#1 "single-blind method"[MeSH Terms] OR "double-blind method"[MeSH Terms] OR "randomized controlled trials as topic"[MeSH Terms] OR "randomized controlled trial" [Publication Type] OR "intention to treat analysis"[MeSH Terms] OR "controlled clinical trials as topic"[MeSH Terms] OR "clinical trials as topic"[MeSH Terms] OR "clinical trial" [Publication Type] OR randomized controlled trial[Publication Type]

#2 random*[Text Word] OR allocation[Text Word] OR "random allocation"[Text Word] OR placebo[Text Word] OR "single Blind"[Text Word] OR "double blind"[Text Word] OR "randomized controlled trial*"[Text Word] OR RCT[Text Word]

#3 #1 OR #2

#4 animals NOT humans

#5 #3 NOT #4

#6  ("pain"[MeSH Terms] OR pain*[Title/Abstract] OR discomfort[Title/Abstract] OR suffering*[Title/Abstract] OR soreness[Title/Abstract] OR analgesia[Title/Abstract] OR ache*[Title/Abstract]) AND ("spine"[MeSH Terms] OR spin*[Title/Abstract] OR "vertebral column"[Title/Abstract] OR "vertebral columns"[Title/Abstract] OR "spinal column"[Title/Abstract] OR "spinal columns"[Title/Abstract] OR vertebra*[Title/Abstract] OR "back"[MeSH Terms] OR back[Title/Abstract] OR "neck"[MeSH Terms] OR neck[Title/Abstract] OR cervical[Title/Abstract] OR cervicothoracic[Title/Abstract] OR lumbar[Title/Abstract] OR "lumbosacral region"[Title/Abstract] OR lumbosacral[Title/Abstract] OR intervertebral[Title/Abstract] OR vertebr*[Title/Abstract] OR coccyx[Title/Abstract] OR sacrum[Title/Abstract] OR sacral[Title/Abstract] OR sacrococcygeal[Title/Abstract] OR thoracolumbar[Title/Abstract] OR thoracic[Title/Abstract])

#7 "scoliosis"[MeSH Terms] OR scolios*[Title/Abstract] OR LBP[Title/Abstract] OR backache[Title/Abstract] OR dorsalgia[Title/Abstract] OR dorsodynia[Title/Abstract] OR notalgia[Title/Abstract] OR neckache*[Title/Abstract] OR cervicalgia*[Title/Abstract] OR cervicodynia*[Title/Abstract] OR lumbago[Title/Abstract] OR "lumbar stenosis" OR "osteoporosis"[MeSH Terms] OR osteoporosis[Title/Abstract] OR "tuberculosis, spinal"[MeSH Terms] OR "tuberculosis, spinal"[Title/Abstract] OR "spinal tuberculos*"[Title/Abstract] OR "intervertebral disc disease"[Title/Abstract] OR "lumbar degenerative changes"[Title/Abstract] OR "lumbar spinal stenosis"[Title/Abstract] OR "lumbar instability"[Title/Abstract] OR "viral infections"[Title/Abstract] OR "scheuermann disease"[Title/Abstract] OR spondylitis[Title/Abstract] OR spondyloarthritis[Title/Abstract]

#8 #6 OR #7

#9 "virtual reality"[MeSH Terms] OR "virtual reality"[Title/Abstract] OR VR[Title/Abstract] OR "simulated environment"[Title/Abstract] OR "augmented reality"[Title/Abstract] OR "simulated reality"[Title/Abstract] OR "virtual simulation"[Title/Abstract] OR "virtual technology"[Title/Abstract] OR "virtual technologies"[Title/Abstract] OR "simulation technology"[Title/Abstract] OR "simulation technologies"[Title/Abstract]

#10 children[Title/Abstract] OR child[Title/Abstract] OR pediatric[Title/Abstract]

#11 (#5 AND #8 AND #9 ) NOT #10

**WOS (262) 2023.11.11**

#1 TS=(parallel OR observational OR cross-sectional OR pre-post OR before-after OR controlled trial* OR random* OR randomi* OR intervention*)

#2 TS=(pain* OR discomfort OR suffering* OR soreness OR analgesia OR ache*)

#3 TS=(spin* OR "vertebral Column*" OR "spinal column*" OR vertebra* OR back OR neck* OR cervical OR cervicothoracic OR lumbar OR "lumbosacral region" OR lumbosacral OR intervertebral OR vertebral OR vertebrae OR coccyx OR sacrum OR sacral OR sacrococcygeal OR thoracolumbar OR thoracic)

#4 #2 AND #3

#5 TS=(scolios* OR cervicalgia OR cervicalgias OR cervicodynia* OR LBP OR backache OR lumbago OR dorsalgia OR dorsodynia OR notalgia OR "lumbar stenosis" OR osteoporosis OR "tuberculosis, spinal" OR "intervertebral disc disease" OR "lumbar degenerative changes" OR "lumbar spinal stenosis" OR "lumbar instability" OR "viral infections" OR "scheuermann Disease" OR "spinal Tuberculos*" OR spondylitis OR spondyloarthritis)

#6 #4 OR #5

#7 TS=("virtual reality" OR VR OR "simulated environment" OR "augmented reality" OR "simulated reality" OR "virtual simulation" OR "virtual technology" OR "virtual technologies" OR "simulation technology" OR "simulation technologies" OR "simulated environment")

#8 TS=(children OR child OR pediatric)

#9 (#1 AND #6 AND #7 ) NOT #8

**Cochrane (278-trials) 2023.11.11**

#1 random*:ti,ab,kw OR allocation:ti,ab,kw OR placebo:ti,ab,kw OR ("single blind"):ti,ab,kw OR ("double blind"):ti,ab,kw OR ("randomized controlled trial*"):ti,ab,kw OR RCT:ti,ab,kw OR ("clinical trial*"):ti,ab,kw

#2 ("randomized controlled trial"):pt OR ("clinical trial"):pt

#3 #1 OR #2

#4 MeSH descriptor: [Pain] explode all trees

#5 pain*:ti,ab,kw OR discomfort:ti,ab,kw OR suffering*:ti,ab,kw OR soreness:ti,ab,kw OR analgesia:ti,ab,kw OR ache*:ti,ab,kw

#6 #4 OR #5

#7  MeSH descriptor: [Spine] explode all trees

#8 spin*:ti,ab,kw OR ("vertebral column*"):ti,ab,kw OR ("spinal column*"):ti,ab,kw OR vertebra*:ti,ab,kw

#9 MeSH descriptor: [Back] explode all trees

#10 back:ti,ab,kw

#11 MeSH descriptor: [Neck] explode all trees

#12 neck*:ti,ab,kw OR cervical:ti,ab,kw OR cervicothoracic:ti,ab,kw OR lumbar:ti,ab,kw OR ("lumbosacral region"):ti,ab,kw OR lumbosacral:ti,ab,kw OR intervertebral:ti,ab,kw OR vertebral:ti,ab,kw OR vertebrae:ti,ab,kw OR coccyx:ti,ab,kw OR sacrum:ti,ab,kw OR sacral:ti,ab,kw OR sacrococcygeal:ti,ab,kw OR thoracolumbar:ti,ab,kw OR thoracic:ti,ab,kw

#13 (#7 OR #8 OR #9 OR #10 OR #11 OR #12) AND #6

#14 MeSH descriptor: [Tuberculosis, Spinal] explode all trees

#15 MeSH descriptor: [Osteoporosis] explode all trees

#16 scolios*:ti,ab,kw OR backache:ti,ab,kw OR dorsalgia:ti,ab,kw OR dorsodynia:ti,ab,kw OR notalgia:ti,ab,kw OR lumbago:ti,ab,kw OR dorsalgia:ti,ab,kw OR cervicalgia:ti,ab,kw OR cervicalgias:ti,ab,kw OR cervicodynia*:ti,ab,kw OR ("intervertebral disc disease"):ti,ab,kw OR ("lumbar spinal stenosis"):ti,ab,kw OR ("lumbar instability"):ti,ab,kw OR ("viral infections"):ti,ab,kw OR ("scheuermann disease"):ti,ab,kw OR ("spinal tuberculos*"):ti,ab,kw OR ("lumbar stenosis"):ti,ab,kw OR spondylitis:ti,ab,kw OR spondyloarthritis:ti,ab,kw

#17 #14 OR #15 OR #16

#18 #13 OR #17

#19 MeSH descriptor: [Virtual Reality] explode all trees

#20 ("virtual reality"):ti,ab,kw OR VR OR ("simulated environment"):ti,ab,kw OR ("augmented reality"):ti,ab,kw OR ("simulated reality"):ti,ab,kw OR ("virtual simulation"):ti,ab,kw OR ("virtual technology"):ti,ab,kw OR ("virtual technologies"):ti,ab,kw OR ("simulation technology"):ti,ab,kw OR ("simulation technologies"):ti,ab,kw OR ("simulated environment"):ti,ab,kw

#21 #19 or #20

#22 (children OR child OR pediatric):ti,ab,kw

#23 (#3 AND #18 AND #21) NOT #22

**CINAHL (80) 2023.11.11**

S1 MH("random assignment" OR "placebos" OR "placebo effect" OR "single-blind studies" OR "double-blind studies" OR "triple-blind studies" OR "randomized controlled trials" OR "comparative studies" OR "evaluation research" OR "prospective studies" OR "crossover design" OR "prospective studies" OR "clinical trials" OR "clinical trial registry")

S2 TX (random$ OR allocation OR "random allocation" OR placebo$ OR single blind OR double blind OR "randomi?ed controlled trial*" OR "controlled clinical trial*" OR "comparative study" OR "evaluation stud*" OR "follow-up stud*" OR "prospective stud*" OR "cross-over stud*" OR control$ OR prospectiv$ OR volunteer$ OR "RCT" OR "clinical trial*")

S3 PT (randomized controlled trial OR "clinical trial*")

S4 S1 OR S2 OR S3

S5 MH ("pain") OR AB ( pain* OR discomfort OR suffering* OR soreness OR analgesia OR ache*)

S6 MH ("spine" OR "back" OR "neck" OR "sciatica") OR AB( spin* OR "vertebral column*" OR "spinal column*" OR vertebra* OR back OR neck* OR cervical OR cervicothoracic OR lumbar OR "lumbosacral region" OR lumbosacral OR intervertebral OR vertebral OR vertebrae OR coccyx OR sacrum OR sacral OR sacrococcygeal OR thoracolumbar OR thoracic)

S7 S5 AND S6

S8 MH ("osteoporosis" OR "tuberculosis, spinal") OR AB( osteoporosis OR "tuberculosis, spinal" OR scolios* OR dorsalgia OR dorsodynia OR notalgia OR backache OR dorsalgia OR cervicalgia OR cervicalgias OR cervicodynia* OR lumbago OR "lumbar stenosis" OR "intervertebral disc disease" OR "lumbar degenerative changes" OR "lumbar spinal stenosis" OR "lumbar instability" OR "viral infections" OR "scheuermann disease" OR "spinal tuberculos*" OR spondylitis OR spondyloarthritis)

S9 S7 OR S8

S10 MH ("virtual reality") OR AB ("virtual reality" OR VR OR "simulated environment" OR "augmented reality" OR "simulated reality" OR "virtual simulation" OR "virtual technology" OR "virtual technologies" OR "simulation technology" OR "simulation technologies" OR "simulated environment")

S11 AB(children OR child OR pediatric)

S12  (S4 AND S9 AND S10) NOT S11

**Embase (191) 2023.11.11**

#1 'parallel design'/exp OR 'observational method'/exp OR 'cross-sectional study'/exp OR 'randomization'/exp OR 'placebo'/exp OR 'placebo effect'/exp OR 'single blind procedure'/exp OR 'double blind procedure'/exp OR 'randomized controlled trial'/exp OR 'controlled clinical trial'/exp OR 'controlled clinical trial (topic)'/exp OR 'clinical trial'/exp OR 'clinical trial (topic)'/exp

#2 random*:ab,ti OR allocation:ab,ti OR ‘random allocation’:ab,ti OR placebo:ab,ti OR ‘single blind’:ab,ti OR ‘double blind’:ab,ti OR ‘randomised controlled trial*’:ab,ti OR ‘randomized controlled trial*’:ab,ti OR RCT:ab,ti OR ‘clinical trial*’:ab,ti

#3 #1 OR #2

#4 'pain'/exp OR pain:ab,ti OR painful:ab,ti OR pains:ab,ti OR discomfort:ab,ti OR suffering:ab,ti OR sufferings:ab,ti OR soreness:ab,ti OR analgesia:ab,ti OR ache:ab,ti OR aches:ab,ti

#5 'spine'/exp OR spine:ab,ti OR spinal:ab,ti OR 'vertebral column':ab,ti OR 'vertebral columns':ab,ti OR 'spinal column':ab,ti OR 'spinal columns':ab,ti OR vertebra:ab,ti OR vertebras:ab,ti OR 'back'/exp OR back:ab,ti OR 'neck'/exp OR neck:ab,ti OR cervical:ab,ti OR cervicothoracic:ab,ti OR lumbar:ab,ti OR 'lumbosacral region':ab,ti OR lumbosacral:ab,ti OR Intervertebral:ab,ti OR vertebral:ab,ti OR vertebrae:ab,ti OR coccyx:ab,ti OR sacrum:ab,ti OR sacral:ab,ti OR sacrococcygeal:ab,ti OR thoracolumbar:ab,ti OR thoracic:ab,ti

#6 #4 AND #5

#7 scolios*:ab,ti OR 'back pain':ab,ti OR LBP:ab,ti OR backache:ab,ti OR dorsalgia:ab,ti OR dorsodynia:ab,ti OR notalgia:ab,ti OR 'back pain':ab,ti OR LBP:ab,ti OR backache:ab,ti OR dorsalgia:ab,ti OR dorsodynia:ab,ti OR notalgia:ab,ti OR lumbago:ab,ti OR lumbago:ab,ti OR 'lumbar stenosis':ab,ti OR 'osteoporosis'/exp OR osteoporosis:ab,ti OR 'tuberculous spondylitis'/exp OR 'intervertebral disc disease':ab,ti OR 'lumbar degenerative changes':ab,ti OR 'lumbar spinal stenosis':ab,ti OR 'lumbar instability':ab,ti OR 'viral infections':ab,ti OR 'scheuermann disease':ab,ti OR 'spinal tuberculos*':ab,ti OR spondylitis:ab,ti OR spondyloarthritis:ab,ti

#8 #6 OR #7

#9 'virtual reality'/exp OR 'educational virtual realities':ab,ti OR 'educational virtual reality':ab,ti OR VR:ab,ti OR 'simulated environment':ab,ti OR 'augmented reality':ab,ti OR 'simulated reality':ab,ti OR 'virtual simulation':ab,ti OR 'virtual technology':ab,ti OR 'virtual technologies':ab,ti OR 'simulation technology':ab,ti OR 'simulation technologies':ab,ti OR 'simulated environment':ab,ti

#10  #3 and #8 and #9
