# Supplementary material for: Virtual Reality Therapy for the Management of Chronic Spinal Pain: Systematic Review and Meta-Analysis
Source: JMIR Serious Games. 2024 Feb 12;12:e50089. doi: 10.2196/50089 (PMC10897798; doi:10.2196/50089)
Supplement: Multimedia Appendix 3 [file games_v12i1e50089_app3.docx]

3. GRADE

**Author(s):**

**Question:** VR group compared to control group for chronic spinal pain in the adults

**Setting:**

**Bibliography:**

| **Certainty assessment** | | | | | | | **№ of patients** | | **Effect** | | **Certainty** | **Importance** |
| --- | --- | --- | --- | --- | --- | --- | --- | --- | --- | --- | --- | --- |
| **№ of studies** | **Study design** | **Risk of bias** | **Inconsistency** | **Indirectness** | **Imprecision** | **Other considerations** | **VR group** | **control group** | **Relative (95% CI)** | **Absolute (95% CI)** |  |  |
| **pain intensity** | | | | | | | | | | | | |
| 15 | randomised trials | not serious | serious^a^ | not serious | serious^b^ | none | 388 | 388 | - | MD **1.63 lower** (2.11 lower to 1.16 lower) | ⨁⨁◯◯ Low | CRITICAL |
| **Inflammatory Markers(CRP)** | | | | | | | | | | | | |
| 2 | randomised trials | not serious | not serious | not serious | serious^c^ | none | 32 | 32 | - | MD **0.89 lower** (1.07 lower to 0.7 lower) | ⨁⨁⨁◯ Moderate | CRITICAL |
| **Inflammatory Markers(TNF-α)** | | | | | | | | | | | | |
| 2 | randomised trials | not serious | not serious | not serious | serious^c^ | none | 32 | 32 | - | MD **6.6 lower** (8.56 lower to 4.64 lower) | ⨁⨁⨁◯ Moderate | IMPORTANT |
| **Inflammatory Markers(1L-2）** | | | | | | | | | | | | |
| 2 | randomised trials | not serious | not serious | not serious | serious^c^ | none | 32 | 32 | - | MD **3.13 higher** (2.9 higher to 3.36 higher) | ⨁⨁⨁◯ Moderate | IMPORTANT |
| **Inflammatory Markers(1L-4)** | | | | | | | | | | | | |
| 2 | randomised trials | not serious^a^ | not serious | not serious | serious^c^ | none | 32 | 32 | - | MD **18.98 higher** (16.31 higher to 21.65 higher) | ⨁⨁⨁◯ Moderate | IMPORTANT |
| **Inflammatory Markers(1L-6)** | | | | | | | | | | | | |
| 2 | randomised trials | not serious | not serious | not serious | serious^c^ | none | 32 | 32 | - | MD **2.76 lower** (2.98 lower to 2.53 lower) | ⨁⨁⨁◯ Moderate | IMPORTANT |
| **Psychosocial Variables:TSK-11** | | | | | | | | | | | | |
| 2 | randomised trials | not serious | serious^d^ | not serious | serious^c^ | none | 33 | 33 | - | MD **0.81 lower** (4.48 lower to 2.86 higher) | ⨁⨁◯◯ Low | IMPORTANT |
| **Psychosocial Variables:TSK-17** | | | | | | | | | | | | |
| 2 | randomised trials | not serious | serious^a^ | not serious | serious^c^ | none | 48 | 48 | - | MD **9.66 lower** (22.01 lower to 2.68 higher) | ⨁⨁◯◯ Low | IMPORTANT |
| **Disability level** | | | | | | | | | | | | |
| 3 | randomised trials | not serious | serious^d^ | not serious | serious^b^ | none | 69 | 70 | - | MD **2.66 lower** (5.47 lower to 0.15 higher) | ⨁⨁◯◯ Low | IMPORTANT |
| **ROM: Extension** | | | | | | | | | | | | |
| 3 | randomised trials | not serious | serious^e^ | not serious | serious^b^ | none | 68 | 68 | - | MD **3.92 higher** (2.17 lower to 10 higher) | ⨁⨁◯◯ Low | IMPORTANT |
| **ROM: Flexion** | | | | | | | | | | | | |
| 3 | randomised trials | not serious | serious^f^ | not serious | serious^b^ | none | 68 | 68 | - | MD **2.67 higher** (2.31 lower to 7.64 higher) | ⨁⨁◯◯ Low | IMPORTANT |
| **ROM: Left rotation** | | | | | | | | | | | | |
| 3 | randomised trials | not serious | serious^g^ | not serious | serious^b^ | none | 68 | 68 | - | MD **0.08 higher** (3.9 lower to 4.05 higher) | ⨁⨁◯◯ Low | IMPORTANT |
| **ROM:Right rotation** | | | | | | | | | | | | |
| 3 | randomised trials | not serious | serious^h^ | not serious | serious^b^ | none | 68 | 68 | - | MD **0.22 lower** (4.38 lower to 3.95 higher) | ⨁⨁◯◯ Low | IMPORTANT |

**CI:** confidence interval; **MD:** mean difference

#### Explanations

a. The high heterogeneity of the included studies and the lack of overlap between the point estimates and the 95% CL were considered to reduce the degree of inconsistency by one level.

b. The 95% CL is wide and contains invalid values.

c. Small sample size

d. the overlap between the point estimates and 95% CL was not good, so the inconsistency was considered to be reduced by one level.

e. Three literatures were included, with high heterogeneity (65%), and the overlap between the point estimates and 95% CL was not good, so the inconsistency was considered to be reduced by one level.

f. Three literatures were included, with high heterogeneity (61%), and the overlap between the point estimates and 95% CL was not good, so the inconsistency was considered to be reduced by one level.

g. literatures were included with high heterogeneity (42%), and the overlap between the point estimates and 95% CL was not good, so the inconsistency was considered to be reduced by one level.

h. The overlap between the point estimates and 95% CL was not good, so the inconsistency was considered to be reduced by one level.
