# Supplementary material for: Virtual Reality Therapy for the Management of Chronic Spinal Pain: Systematic Review and Meta-Analysis
Source: JMIR Serious Games. 2024 Feb 12;12:e50089. doi: 10.2196/50089 (PMC10897798; doi:10.2196/50089)
Supplement: Multimedia Appendix 4 [file games_v12i1e50089_app4.docx]

4. Multimedia Appendix


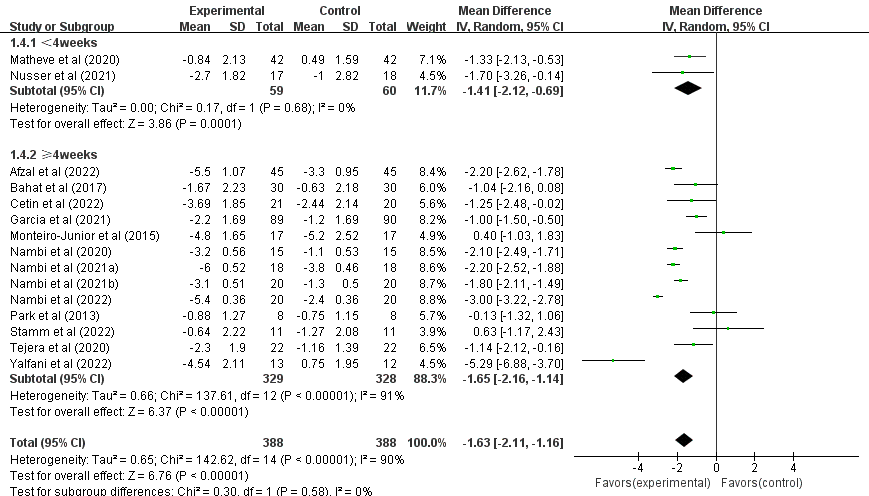


***S1. Forest plots of the effect of virtual reality compared with other treatments for pain intensity in patients of chronic spinal pain -- subgroup analysis of posttreatment effectiveness for treatment duration.***
