# Supplementary material for: Virtual Reality Therapy for the Management of Chronic Spinal Pain: Systematic Review and Meta-Analysis
Source: JMIR Serious Games. 2024 Feb 12;12:e50089. doi: 10.2196/50089 (PMC10897798; doi:10.2196/50089)
Supplement: Multimedia Appendix 5 [file games_v12i1e50089_app5.docx]

5. Multimedia Appendix

**Secondary Outcomes**

***1. Inflammatory Markers***

*(a) CRP*


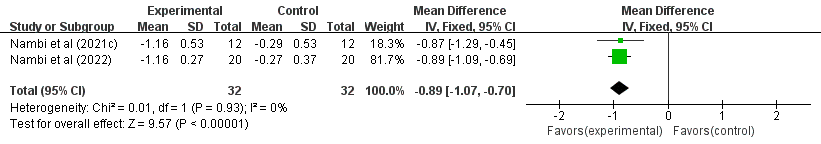


*(b) TNF-α*
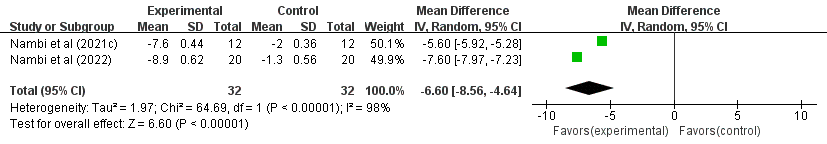


*(c) 1L-6*
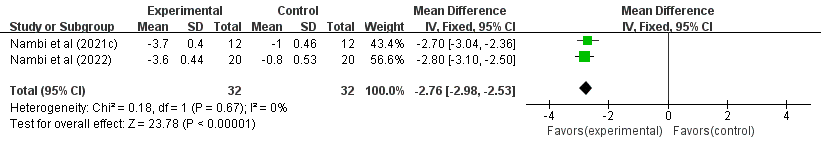


*(d) 1L-2*
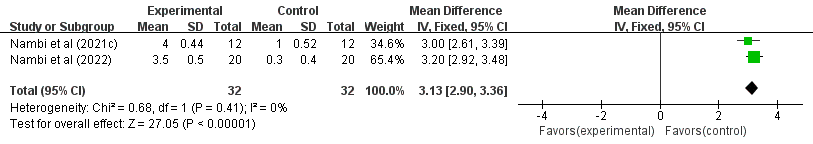


*(e) 1L-4*
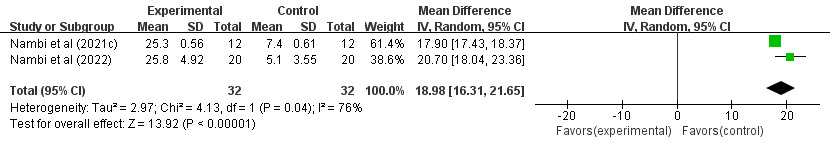
 *CRP: C-reactive protein, TNF-α: tumor necrosis factor-alpha, 1L-6: interleukins 6, 1L-2: interleukins 2, 1L-4: interleukins 4.*

***S1. Forest plots of the virtual reality compared with other treatments for inflammatory markers levels in patients of chronic spinal pain. (a) CRP; (b) TNF-α; (c) 1L-6; (d) 1L-2; (e) 1L-4.***

***2. Psychosocial Variables***


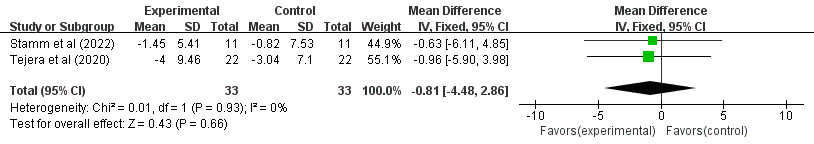


*TSK-11: the 11-items Tampa Scale of Kinesiophobia, TSK-17: the 17-items Tampa Scale of Kinesiophobia.*

***S2. Forest plots of the effect of virtual reality on the psychosocial variables measured by TSK-11 in patients of chronic spinal pain.***


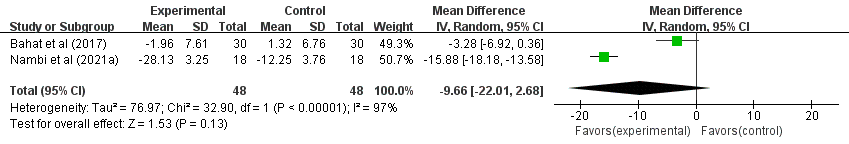


***S3. Forest plots of the effect of virtual reality on the psychosocial variables measured by TSK-17 in patients of chronic spinal pain.***

***3. Spinal range of motion***

*(a)* *Flexion*


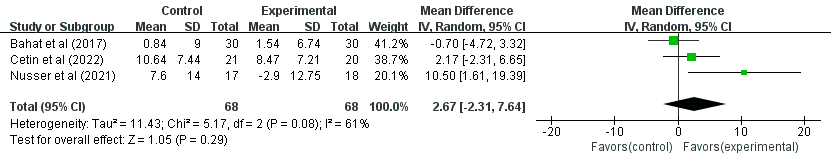


*(b)* *Extension*


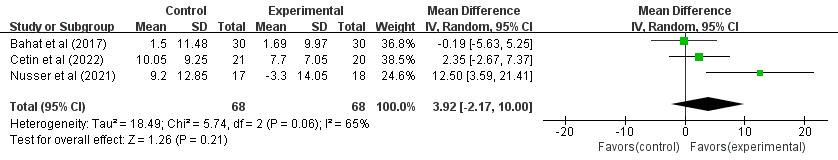


*(c)* *Right rotation*


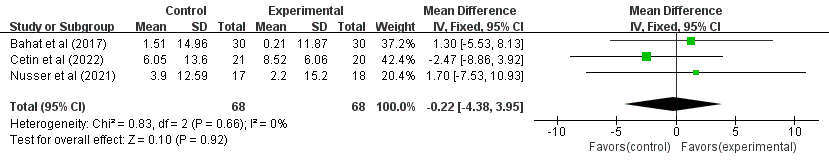


*(d)Left rotation.*
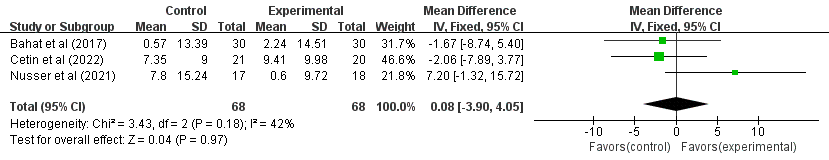


*ROM: range of motion*

***S4. Forest plots of the effect of virtual reality compared with other treatments for spinal ROM in patients of chronic spinal pain. (a)*** ***Flexion; (b)*** ***Extension;*** ***(c)Right rotation; (d)Left rotation.***

***4. Disability level***


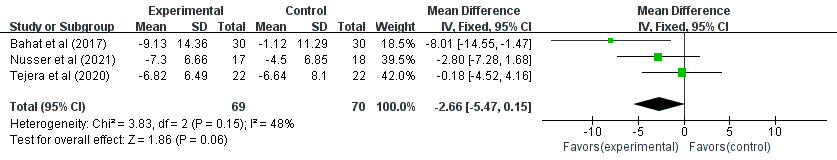


*NDI: Neck disability Index*

***S5. Forest plots of the effect of virtual reality compared with other treatments on disability measured by the NDI*** ***in patients of chronic spinal pain.***
